# Targeting Brain Drug Delivery with Macromolecules Through Receptor-Mediated Transcytosis

**DOI:** 10.3390/pharmaceutics17010109

**Published:** 2025-01-15

**Authors:** Yuanke Li, Ruiying Liu, Zhen Zhao

**Affiliations:** 1 State Key Laboratory of Medicinal Chemical Biology, Key Laboratory of Bioactive Materials for the Ministry of Education, College of Life Sciences and Frontiers Science Center for Cell Responses, Nankai University, Tianjin 300071, China; 2 National Key Laboratory of Intelligent Tracking and Forecasting for Infectious Diseases, TEDA Institute of Biological Sciences and Biotechnology, Nankai University, Tianjin 300457, China; 3 Key Laboratory of Molecular Biophysics, Institute of Biophysics, School of Health Sciences & Biomedical Engineering, Hebei University of Technology, Tianjin 300401, China

**Keywords:** receptor-mediated transcytosis, CNS, ligands, antibody, peptide, aptamer, drug delivery

## Abstract

Brain diseases pose significant treatment challenges due to the restrictive nature of the blood–brain barrier (BBB). Recent advances in targeting macromolecules offer promising avenues for overcoming these obstacles through receptor-mediated transcytosis (RMT). We summarize the current progress in targeting brain drug delivery with macromolecules for brain diseases. This exploration details the transport mechanisms across the BBB, focusing on RMT and its use of natural ligands for drug delivery. Furthermore, the review examines macromolecular ligands such as antibodies, peptides, and aptamers that leverage RMT for effective BBB traversal. Advancements in macromolecules-based delivery systems for brain diseases are summarized, emphasizing their therapeutic potential and limitations. Finally, emerging RMT strategies, including viral vectors, exosomes, and boron neutron capture therapy, are discussed for their precision in brain-targeted treatments. This comprehensive overview underscores the potential of RMT-based approaches to revolutionize brain disease therapy.

## 1. Introduction

Brain diseases, including central nervous system (CNS) disorders and brain cancers, are some of the most common and devastating health issues, yet they are often treated inadequately. Typically, developing CNS drugs takes significantly longer than developing non-CNS drugs [1]. Clinical trials for CNS drugs are particularly challenging due to the complexity of the brain, potential side effects, and the difficulties posed by the blood–brain barrier (BBB) [2]. According to a study data released by *Lancet Neurology* in 2024, over 3 billion people worldwide were living with a neurological condition [3]. In another report from the American Cancer Society, over 25,000 new cases of malignant brain or spinal cord tumors are diagnosed annually in the United States, with the incidence continuing to rise rapidly [4]. In contrast, there are currently limited therapeutic options available on the market to treat brain diseases, summarized in Table 1. Macromolecular drugs have rapidly developed in recent years because of their high specificity and prolonged blood circulation time. These macromolecules have been successfully applied to treat various diseases, including CNS disorders and brain cancers.

In 2021, aducanumab, a human immunoglobulin gamma 1 (IgG1) monoclonal antibody (mAb) to clean amyloid β (Aβ), became the first approved treatment for Alzheimer’s disease (AD) via the Food and Drug Administration (FDA) since 2003 [5]. However, its manufacturers discontinued the AD-related mAb from clinical application in January 2024 [6]. Later, in 2023, lecanemab, another Aβ-targeting monoclonal antibody, was approved to treat Alzheimer’s in patients with mild cognitive impairment or mild dementia [7]. Beyond the two well-known AD-targeting macromolecular antibodies, bevacizumab, a vascular endothelial growth factor A (VEGF-A)-targeting monoclonal antibody approved in 2004 for metastatic colorectal cancer, was later approved to treat glioblastoma [8]. Dinutuximab, targeting disialoganglioside GD2, received FDA approval in 2015 for combination therapy in high-risk neuroblastoma in pediatric patients [9]. GD2 is a tumor-associated surface antigen with significantly higher expression in tumor cells, making it an ideal target for diagnosis and therapy [10]. More recently, naxitamab, another anti-GD2 monoclonal antibody, gained accelerated FDA approval as a treatment for pediatric patients in 2020 [11]. Although there are currently no approved peptide- or aptamer-based drugs for treating brain diseases, their shared characteristics with antibodies—such as specific target recognition and stable circulation—make it foreseeable that related therapeutics will soon enter the public spotlight.

Unlike small-molecule drugs, which can cross the BBB relay via their physicochemical properties (lipid-soluble) and small molecular weight (less than 400–500 Da), macromolecules such as antibodies, peptides, and aptamers are limited by their larger size and cannot penetrate the BBB directly [12]. However, these macromolecules can specifically bind to brain capillary endothelial cells (BCECs), enabling their effective accumulation in the brain. Moreover, they can act as targeted shuttles, facilitating precise drug delivery to the brain through strategies such as covalent conjugation or drug encapsulation [13,14,15]. These targeted approaches enhance the therapeutic potential of macromolecules in treating brain disorders by ensuring that higher concentrations of the therapeutic agents reach the intended site of action within the brain while minimizing systemic exposure and potential side effects [16,17]. In this review, we summarize the current progress in targeting brain drug delivery with macromolecules for brain diseases via RMT, including the natural ligands of these RMT receptors, as well as designed macromolecular ligands such as antibodies, peptides, and aptamers for effective BBB traversal.

## 2. Transport Mechanisms Cross the Blood–Brain Barrier

**Figure 1:**
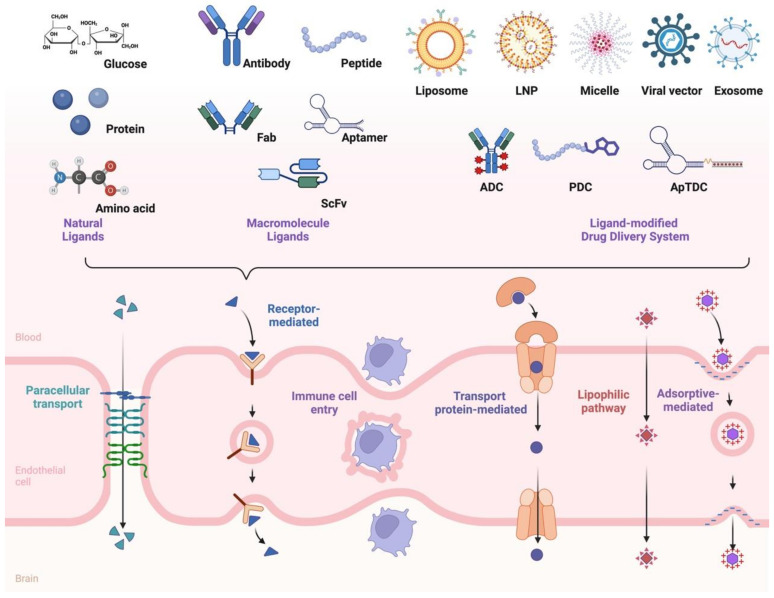
Transport mechanisms at the blood–brain barrier and ligand-mediated drug delivery. Small molecules can enter the brain via the paracellular pathway. Transcellular passage across BCECs can be divided into the lipophilic pathway, the leukocyte cell entry route, the transport protein pathway, adsorptive transcytosis, and receptor-mediated transcytosis. Natural ligands, including glucose, proteins, amino acids, and macromolecule ligands (antibody, Fab, ScFv, peptide, and aptamer), can interact with receptors on the surface of BCECs and imitate the multi-step receptor-mediated endocytosis. Such ligands can be further modified to drug delivery systems for treating CNS diseases (Illustration by Biorender).

The BCECs form a protective blood–brain barrier that selectively regulates the movement of substances between the bloodstream and the central nervous system, shielding the brain from harmful or unwanted chemicals [18]. Only small molecules that are lipid-soluble and have a molecular weight of less than 400–500 Da can effectively cross the BBB, and most macromolecules cannot penetrate the brain’s endothelium directly [12]. In addition to small molecules trailing the paracellular penetration pathway, lipophilic compounds can cross the barrier primarily through transmembrane diffusion. This process involves the drug molecules diffusing directly through the cell membranes of the BCECs that constitute the BBB [19]. Most small molecular drugs cross the BBB via either the paracellular penetration pathway or transmembrane diffusion. This non-saturable diffusion requires a significant accumulation of the drug around the BCECs, which increases the risk of systemic side effects and toxicity. While the high lipid solubility of these drugs enhances their ability to diffuse across membranes, it can also lead to unexpected accumulation in peripheral tissues [19,20]. The transcellular passage through the BCECs involves the movement of substances directly through the cell membrane [17]. This pathway is essential for maintaining the selective permeability of the BBB and ensuring that vital nutrients and molecules can access the brain while keeping harmful substances out [17,21]. Such transcellular approaches include the mentioned lipophilic pathway, the leukocyte cells entry route, the transport protein pathway, adsorptive transcytosis, and receptor-mediated transcytosis (Figure 1) [22].

Under normal conditions, the blood–brain barrier is highly selective and restricts the entry of leukocytes to protect the brain. However, the BBB becomes more permeable during inflammation or injury, allowing leukocytes to pass through [23]. Inflammatory signals, such as cytokines, including tumor necrosis factor-alpha (TNF-α) and interleukins, can loosen the tight junctions between endothelial cells, facilitating the migration of leukocytes across the barrier [24]. Additionally, leukocytes can traverse the barrier using the transcellular pathway, which involves passing directly through the endothelial cells. This process includes the formation of transient pores in the membranes of endothelial cells by the leukocytes [25]. Leukocytes can pass through the epithelial cells of the choroid plexus, which is responsible for producing cerebrospinal fluid, or migrate through the blood vessels in the meninges [26,27]. In past decades, drug delivery systems have been designed to mimic leukocytes to enhance delivery across the blood–brain barrier [28].

Transport proteins provide an alternative pathway for drugs to cross BCECs [29]. One key transport protein involved in this process is P-glycoprotein (P-gp), which plays a significant role in the transport of drugs across the BBB [30]. P-gp is primarily found on the luminal (apical) surface of the BCECs that compose the BBB. Levodopa (L-DOPA, also known as l-3,4-dihydroxyphenylalanine) is a prodrug for the treatment of Parkinson’s disease that is converted into dopamine through the action of decarboxylases [31]. Levodopa is recognized as a substrate for an amino acid transport protein known as the large amino acid transporter-1 (LAT1). This transporter is highly expressed in the BCECs found at the luminal and abluminal membranes [31]. However, as the multidrug resistance-associated membrane protein, P-gp functions as efflux transporters, utilizing ATP to pump drugs and other substances out of the brain and thereby protecting it from potentially harmful compounds [30,32].

In addition to transport proteins, another important class of membrane proteins facilitates macromolecular transcytosis across the blood–brain barrier and generates the receptor-mediated transcytosis pathway, which is used to deliver therapeutic antibodies and other macromolecules to the brain [33]. Receptor-mediated transport (RMT) is a multi-step process that involves the receptor-mediated endocytosis (RME) of macromolecules on the surface of BCECs. These macromolecules are then sorted within endosomes and eventually exocytosed at the opposite side of BCECs, completing the BBB-crossing progress.

## 3. Receptor-Mediated Transports and Natural Ligands

### 3.1. Transferrin Receptor 1

Transferrin receptor 1 (TfR1, CD71) is a transmembrane glycoprotein involved in transferrin-mediated iron uptake [34]. It is highly expressed on the surface of BCECs and is widely used as a BBB penetration drug delivery target [33]. The natural ligand of TfR1 is transferrin (Tf), an 80 kDa glycoprotein that can transport two Fe^3+^ ions at a time through RME upon binding to TfR1 with a nanomole-level affinity [35,36]. Due to a high-fold difference in binding affinity to TfR2, Tf-coupled drugs exhibit enhanced tissue distribution and blood–brain barrier penetration [34,35]. Drugs like doxorubicin and cisplatin were covalently linked to Tf directly for anti-cancer therapy in the early 21st century [37]. Immunotoxins are also linked to Tf to enhance their binding affinity and BBB penetration ability. Tf-CRM107 is a mutant form of diphtheria toxin that contains two amino acid mutations in the B chain and conjugated with human Tf [38]. However, a Phase III multicenter study of Tf-CRM107 for glioblastoma was withdrawn for the unexpected efficacy (NCT00083447). Research explores the coupling of Tf with nanocarriers, including liposomes and organic/inorganic nanoparticles, based on the Tf/TfR1 complex for treating brain diseases [35,39,40,41]. Another crucial natural ligand for TfR1 is ferritin [42,43]. Ferritin is a 474 kDa protein found both inside and outside cells, and it is responsible for storing iron and releasing it in a controlled manner [44]. Nearly all living organisms produce it, and it serves as the primary iron-storage protein in both prokaryotes and eukaryotes, maintaining iron in a soluble and non-toxic form. The protein is a globular complex comprising 24 subunits that form a hollow, spherical nanocage with multiple interactions between metals and proteins [45]. The 12-nm external and 8-nm inner diameter spherical structure offer native ferritin the ability to carry drugs to cross the BBB via TfR1 [34,46].

### 3.2. Large Neutral Amino Acid Transporter Type 1

The large neutral amino acid transporter type 1 (LAT1) is a membrane transport protein complex consisting of two glycoproteins, SLC3A2 and SLC7A5. LAT1 is a Na^+^ and pH-independent exchanger, and it primarily facilitates the transport of branched-chain amino acids (valine, leucine, and isoleucine) and aromatic amino acids (tryptophan, tyrosine, and phenylalanine). It is highly expressed in the brain capillaries that constitute the blood–brain barrier, making it more prevalent in the CNS than in other tissues [47,48]. The high expression of the LAT1 transporter on both the luminal and abluminal surfaces of BCECs, along with its presence on brain parenchymal cells, highlights its promising role in CNS-targeted drug delivery [49]. LAT1 has effectively facilitated the transport of several clinically used CNS drugs and prodrugs—such as L-DOPA, baclofen, alpha-methyldopa, and gabapentin—across the BBB [48]. This underscores its potential as a critical transport mechanism for therapeutics targeting the CNS. The LAT1 transporter, restricted by its primary function as an amino acid transporter, is inherently limited to carrying small-molecule drugs conjugated to natural amino acids or similar structure molecules across BCECs. The LAT1 binding site is believed to consist of three distinct recognition sites: a positive site, a negative site, and a hydrophobic site [50]. These sites are responsible for recognizing and binding the negatively charged α-carboxyl group, the positively charged α-amino group, and the side chain of the amino acid, respectively. This configuration allows LAT1 to transport branched-chain and aromatic amino acids. The most successful LAT1-based CNS drug is L-DOPA for Parkinson’s disease [31]. The L-DOPA with the structure as l-3,4-dihydroxyphenylalanine can easily bind to the recognition sites in LAT1 and internalize the LAT1-mediated transport on BCECs [50]. A novel chemical entity (QBS10072S) has been developed for delivering a cytotoxic chemotherapeutic domain to GBM cells via LAT1 with a 50-fold higher selectivity than LAT2 [51]. Other small molecule-based LAT1-mediated BBB delivery strategies have been reviewed [48].

LAT1 plays a vital role in maintaining normal physiological functions by regulating the exchange of amino acids in synthesizing peptides, proteins, neurotransmitters, and nutrient metabolism. Therefore, when designing LAT1-mediated drug delivery systems to cross the BBB, it is essential to ensure that the transporter’s critical functions remain unaffected to avoid disrupting these fundamental metabolic processes [48]. CD98 is a glycoprotein composed of SLC3A2 in LAT1 and its heavy subunit protein encoded by the *SLC3A2* gene [52]. Chew et al. identified that the CD98hc is a target for brain delivery and further developed a BBB transport vehicle (ATV^CD98hc^) [53]. The ATV^CD98hc^ performed an enhanced brain uptake in cynomolgus monkeys with unique kinetics and biodistribution properties [53]. A CD98-ScFv-based bispecific antibody shuttle has also been recently developed by Pornnoppado et al. to cross the BBB [54]. The CD98hc-mediated shuttling results in the prolonged retention of immunoglobulins (IgGs) in the brain compared to TfR1-mediated shuttling. This enhanced retention is achieved through limited engagement of CD98hc within the brain parenchyma, thereby enabling more precise brain targeting [54]. Interestingly, beyond the two CD98hc-targeting antibodies, none of the other Fab, ScFv, nanobodies, peptides, or aptamers have been developed as shuttle ligands based on the LAT1-related RMT. One possible reason may be the thriving small molecular-based BBB shuttle strategies in the past decades [48]. Another implicit factor may be the recent understanding of the structure of the human LAT1-related complexes [55,56]. An increasing number of LAT1-targeting macromolecular shuttle ligands are expected to be developed in the near future.

### 3.3. Glucose Transporter 1

GLUT1, a glucose transporter 1- or solute carrier family 2-facilitated glucose transporter member 1 (SLC2A1), is a uniporter protein encoded by the human *SLC2A1* gene [57]. GLUT1 is a member of the sugar porter subfamily within the major facilitator superfamily (MFS), a highly conserved and widespread group of secondary transporters [58]. GLUT1 is pivotal in mediating glucose transport across mammalian cells’ plasma membranes and controlling glucose uptake and glycolysis in endothelial cells [59]. The *SLC2A1* gene encodes this facilitative glucose transporter, which is highly expressed in erythrocytes and endothelial cells, including BCECs [59]. The stereochemical properties of GLUT1 facilitate the binding of both D-glucose and D-galactose [60]. The hydroxyl group of glucose at the C-6 position interacts with a hydrophobic pocket within the substrate-binding site of the transporter protein [61]. Utilizing the natural property of GLUT1 as the sensitizer for glucose, ketoprofen-based and indomethacin-based prodrugs have been designed and synthesized with enhanced rat brain accumulation [62]. Zhao et al. designed a lipophilic thiamine disulfide system to conjugate ibuprofen with L-ascorbic acid, incorporating a “lock-in” mechanism to form a prodrug [63]. This L-ascorbic acid-derived molecule demonstrated exceptional BBB permeability, facilitated via GLUT1 and the sodium-dependent vitamin C transporter SVCT2. The same research group further developed a liposomal delivery system incorporating L-ascorbic acid, which enhanced the brain concentration of docetaxel by 5.62-fold [64]. Relying on the same GLUT1 transporter, a galactose-modified nanodelivery system was developed to transport BACE1 siRNA across the blood–brain barrier (BBB), effectively reducing BACE1 expression in APP/PS1 transgenic mice [65]. While GLUT1 demonstrates the highest transport capacity among carrier-mediated transporters at the BBB [62], its limitations include an inability to transport large molecules and competition between glucose and therapeutic agents, which may compromise drug delivery efficiency. Excessive loading of therapeutic agents on this transporter could further disrupt regular glucose transport, potentially causing metabolic imbalances [66,67].

### 3.4. Low-Density Lipoprotein Receptor-Related Protein (LRP)

LRP1, also known as CD91, is a plasma membrane receptor involved in receptor-mediated endocytosis. Encoded by the *LRP1* gene, it plays key roles in signaling, lipoprotein metabolism, and cell motility, and it is linked to diseases like neurodegenerative disorders, atherosclerosis, and cancer [68,69]. As part of the LDL receptor family, LRP1 mediates ligand internalization for lysosomal degradation and supports neuronal cholesterol uptake for membrane stability [70]. It is synthesized as a 600 kDa precursor protein and cleaved by Furin in the Golgi complex into a 515 kDa extracellular α-chain with four ligand-binding domains and an 85 kDa β-chain [71]. LRP1 consists of an extracellular domain (ECD) that binds over 30 ligands, including ApoE (apolipoprotein E), α2-macroglobulin, tPA (tissue plasminogen activator), proteinase inhibitors, coagulation factors, RAP (receptor-associated protein), Aβ, prion protein, and aprotinin and an intracellular domain (ICD) that interacts with signaling proteins, often acting as co-receptors [70]. The LRP1 acts as a master regulator of tau protein propagation and aggregation for the development of Alzheimer’s disease in the brain [72]. Further studies have identified LRP1 as a neuronal receptor for α-synuclein (α-Syn) uptake and spread, contributing to the pathology of Parkinson’s disease and Lewy body dementia [73]. By mimicking LRP1’s transport function across BCECs, a German research group developed a truncated mini LRP1 (mLRP1_DIV*) as a liposomal carrier to cross the BBB and deliver the γ-secretase modulator BB25 from luminal to basolateral side, reducing toxic Aβ42 peptide expression [74].

LRP8, also known as apolipoprotein E receptor 2 (ApoER2), is an LDLR family member involved in transporting lipoproteins and other molecules across the BBB via RMT [75]. It is a single-pass transmembrane protein with an extracellular domain featuring seven LDLR Class A repeats, three EGF repeats integrated with a β-propeller, and an O-linked glycosylation region [75]. LRP8 is primarily recognized as a receptor for ApoE, a lipoprotein component produced in organs such as the liver, brain, kidneys, and adrenal glands [76]. LRP8 binds ApoE-rich β-migrating VLDL with high affinity but shows significantly lower affinity for LDL and other VLDL particles [77]. ApoE facilitates the transport of cholesterol and fats through the bloodstream [76]. Reelin is another ligand for LRP8, as well as the ligand for VLDL receptor [78]. Once binding with LRP8, reelin can regulate the processing of amyloid precursor protein (APP) and the following production of Aβ [79]. Other natural ligands targeting LRP8 in the CNS, including F-Spondin, thrombospondin-1, clusterin, and selenoprotein P, have been detailed in a previous review [80].

### 3.5. Insulin-like Growth Factor (IGF) Receptor (IGFR) 

Insulin-like growth factor receptors include two members: the IGF1 receptor (IGF1R) and the IGF2 receptor (IGF2R). The insulin family consists of insulin, IGF1, IGF2, insulin receptors (IRs), IGF1R, IGF2R, and their binding proteins (IGFBP1 to IGFBP7) [81]. The IGF axis centers on IGF-1, which is predominantly produced by the liver in response to growth hormone (GH). IGF1 is key in regulating normal physiology and contributes to pathological processes like cancer by enhancing cell proliferation and suppressing apoptosis [82]. Meanwhile, IGF2 is more enrolled in embryonic development, metabolic disorders, and tumorigenesis [83]. IGFs interact with multiple receptors, including the IGF1R, IR, IGF2R, and insulin-related receptors. The IGF1R is the primary receptor, with a much higher affinity for IGF1 than IR. Unlike these, the IGF2R binds only IGF2 and acts as a clearance receptor, sequestering IGF2 without triggering intracellular signaling [83].

Both IGF1R, IGF2R, and IR are involved in the RMT on BBB [84]. The IR and IGF1R, both receptor tyrosine kinases, are primarily activated by their specific ligands but can also interact with non-cognate ligands [85]. IR regulates glucose metabolism, and IGF1R promotes cell growth via similar PI3K and MAPK pathways. In contrast, IGF2R binds IGF2 and mannose 6-phosphate (M6P) without signaling and instead facilitates ligand transport to endosomal/lysosomal compartments [85]. IGF1 is critical in neural development, including neurogenesis, myelination, synaptogenesis, dendritic branching, and neuroprotection following neuronal injury [86]. As the primary ligand for IGF1R, a phase I study of an IGF–methotrexate conjugate in treating advanced tumors expressing IGF1R, including brain cancer, has been set and completed (NCT02045368). IGF2 is essential for memory and cognition in adult rodents, with its dysregulation linked to CNS diseases [87]. Both IGF1 and IGF2 can be transported into the brain via IGFR-related RMT. Mucopolysaccharidosis type IIIB (MPS IIIB) is a severe, untreatable disorder primarily impacting the brain [88]. A fusion protein combining recombinant α–*N*-acetylglucosaminidase (NAGLU) with an IGF2 fragment was designed for endocytosis via the IGF2R [88]. The NAGLU–IGFII fusion protein was delivered intracerebroventricularly to bypass the BBB and was taken up via neurons. Notably, NAGLU levels were found to be five times higher in the liver than in the brain, possibly due to the fusion protein mixing with cerebrospinal fluid and entering systemic circulation via blood or lymph [88]. The transport of IGF2/M6P via IGF2R diminishes progressively from infancy to adulthood, with the BBB losing this capacity entirely by adulthood. This suggests that the IGF2R system may be effective only for delivery in neonates [89]. Another challenge for IGF1/2-based BBB delivery is the presence of IGF-binding proteins (IGFBPs) in the plasma. Most IGFs are bound to IGFBPs, forming binary or ternary complexes. About 75–80% are in a ternary complex with IGFBP-3 (or IGFBP-5) and the acid labile subunit (ALS), while the rest are in other IGFBP complexes [90].

### 3.6. Scavenger Receptor (SR)

SR is a heterogeneous family containing SR class A (SR-A, CD204), SR-B1, and SR-B2 (CD36) of cell surface receptors that recognize and internalize various ligands, such as modified lipoproteins, bacteria, and apoptotic cells. These receptors are essential for maintaining cellular homeostasis and defense against harmful substances [91,92]. SRs are capable of binding and internalizing modified LDL forms like acetylated or oxidized LDL, but not native LDL. The functional loss of these SRs can lead to hypercholesterolemia, which contributes to the development of atherosclerosis and heart disease [93]. The high-density lipoprotein (HDL) and HDL-associated α-tocopherol cross the BBB by being taken up and transported via BCECs through SR-B1 [94]. The SR-B2 is expressed in BCECs and contributes to the selective uptake of HDL-associated vitamin E [95]. In 2020, a biomimetic nanoparticle was engineered to target SR-B1 and CD15 for crossing the BBB. This design incorporated apolipoprotein A1 and anti-CD15 to facilitate delivery for treating sonic hedgehog (SHH) subtype medulloblastoma [96]. Mimicking the natural properties of HDL, the nanoparticles utilized SR-B1-mediated receptor-mediated transcytosis (RMT) to cross the BBB, enhancing the activity of LDE225, a SHH pathway inhibitor, in SHH medulloblastoma cells [96]. In another study, a peptide-based vector modified with the targeting ligand Angiopep-2 was designed to deliver plasmid DNA across the BBB via LRP-1. Interestingly, the vector/DNA nanocomplex was transported into BCECs not only through LRP-1 but also via SR-A and SR-B1-mediated RMT [97].

## 4. Macromolecular Ligands for RMTs to Cross BBB

Beyond the natural protein ligands targeting the transcellular pathway enrolled receptors, macromolecules like antibodies, nanobodies, aptamers, and peptides have also been developed for CNS-related drug delivery.

### 4.1. Antibody

TfR1 monomer consists of a large extracellular C-terminal domain with 671 amino acids, which includes the transferrin (Tf) binding site, a 28-amino acid transmembrane domain, and an N-terminal intracellular domain with 61 amino acids. The C-terminal extracellular domain has three essential N-linked glycosylation sites and an O-linked glycosylation site [36]. Several anti-TfR1 antibodies have been developed and can be divided into antagonistic and non-antagonistic antibodies [98]. Phage display technology is a powerful tool to screen the potential binding molecules, including antibodies, ScFvs, nanobodies, and peptides, to the specific protein [99]. Screened binding molecules are primarily functionalized as ligand mimetics, but some exhibit biological activities due to different strategies. Due to the enrolled pathology role in cancer cells, antagonistic antibodies are mainly used to target TfR1 as anti-cancer agents directly [98]. Focused on the brain-related glioblastoma multiforme (GBM), murine anti-human TfR1 IgG antibody 7579 has the ability to inhibit the proliferation of human glioma cells [100]. Non-antagonistic anti-TfR1 antibodies were used more to carrier nanodrug across the BBB via TfR1-mediated RMT while causing no influences on the Tf/TfR1 combination [34]. Antibody-drug conjugates (ADCs) have been developed and approved for the clinic in recent decades [101]. In 2023, an anti-TfR1 antibody-based ADC drug JR141 was approved in Japan to treat Hunter syndrome [102]. A novel iduronate-2-sulfatase fused with a TfR1 mAb based on the technology called J-Brain Cargo^®^ can penetrate BBB and treat mucopolysaccharidosis II (MPS II) efficiently (NCT03128593) [103]. OX26 is an anti-TfR1 IgG monoclonal mAb. The conjugation of the OX26 with a neuropeptide (Galanin) performed an enhanced brain and cerebrospinal fluid exposure and prolonged pharmacokinetics property in a rat model [104]. Another bispecific mAb attempt is confusing two mAb together against TfR and β-secretase to cross the BBB and reduce the level of amyloid β in the brain [105]. High-binding TfR mAb improves brain exposure to anti-BACE1 mAb, and the bispecific mAb significantly inhibited the level of BACE1 [105]. Argiridai et al. developed LRP8-specific antibodies, including 11H1, using cyclic peptide antigens to cross the BBB. A cyclized peptide immunogen was designed to preserve the β-hairpin structure of the LRP8 CR domain. The 11H1 antibody, characterized through ligand-binding assays and crystallography, showed enhanced brain accumulation by binding the LRP8 CR1 domain to form a transporter ternary complex [106].

Performing a similar binding ability, the mAb fragment, including single-chain (sc) Fab fragment, single-chain fragment variable (ScFv), and variable heavy chain (VHH, also known as nanobody), has attracted more and more interest for BBB-related drug development [107]. An anti-TfR1 Fab bound to the apical domain of TfR was chosen and fused to the C-terminal of the heavy chain from anti-Aβ mAb (mAb31) as the monovalent molecular brain shuttle to increase brain penetration and potency [108]. In a PS2APP double-transgenic amyloidosis model, one (sFab) or two (dFab) fused bispecific mAb were injected via i.v. injection, and only sFab-fused mAb31 was found to have a massive increase in plaques. Meanwhile, dFab-mAb31 was only determined in brain microvessels, possibly due to the failure disconnection with TfR at the abluminal side [108]. An anti-TfR1 ScFv8D3 was conjugated with a recombinant humanized anti-Aβ protofibrils mAb158 (BAN2401) to generate a bispecific mAb [109]. The recombinant fusion protein (RmAb158-scFv8D3) markedly increased the brain accumulation of the mAb158 with a 9-fold higher after three days of administration in the old tg-ArcSwe mice model [109].

A particular ScFv-recombinant antibody against SR-B1 was developed for imaging native SR-B1 in various live cells [110]. Using the ScFv’s specific affinity, it was observed that SR-B1 remains retained at the plasma membrane for several hours. This retention is attributed to extensive SR-B1 multimerization, which prevents receptor endocytosis. Disrupting this multimerization impairs HDL binding and compromises SR-B1’s function at the cell membrane [110].

Nanobodies are antigen-binding fragments derived from heavy chains, with a significantly smaller size than traditional monoclonal antibodies (mAbs) or Fab fragments [111]. Recently, Wouter et al. developed a brain-penetrating anti-transferrin receptor (anti-TfR) nanobody, Nb62, which utilizes the hypothermic effect of neurotensin as a readout for CNS target engagement [112]. The anti-mouse TfR nanobody Nb62 crosses the BBB through RMT. This process involves targeting neurotensin receptor 1 (NTSR1) in hypothalamic neurons. Upon successful CNS entry, nanobodies conjugated to neurotensin activate NTSR1, resulting in a quantifiable reduction in body temperature [112]. An anti-human TfR nanobody Nb188 was further developed and conjugated to a BACE1 1A11 Fab to generate a heterodimeric antibody (1A11AM-Nb188) [113]. The systemic administration of 1A11AM-Nb188 led to a significant reduction in brain Aβ levels, and pharmacokinetic and pharmacodynamic (PK/PD) analysis revealed that this Aβ reduction effect could be sustained for up to 3 days post-administration [113]. A similar strategy was performed by fusing an anti-TfR nanobody with anti-Aβ ScFv3D6 [114].

Grabody B, an anti-IGF1R antibody developed by the Lee group, mediates the efficient delivery of biologics as a BBB shuttle [115]. In a Parkinson’s disease animal model, the Grabody B-fused anti-α-synuclein antibody outperforms the therapeutic antibody alone, offering better neuropathological and behavioral improvements due to improved serum pharmacokinetics and increased brain exposure [115]. Targeting cysteine-rich region (CRR) of IGF1R, a BBB-crossing single-domain antibody (sdAb) named VHH-R4 was developed [116]. The VHH-IR4 sdAb inhibits the ligand-induced autophosphorylation of IGF1R through downstream conformational changes [116]. Another sdAb (VHH-IR5) shares the same binding site with IGF1 at the α-CT helix of IGF2R and also performed a BBB transport ability [117]. The same research group also developed three more sdAbs (IGF1R3, IGF1R4, and IGF1R5) to target the ECD of human IGF1R [118]. These sdAbs further fused with mouse Fc (sdAb-mFc) and exhibited an enhanced transmigration ability with a 2-fold fluorescence intensity and a 4-fold distribution volume in the brain [118]. Such novel sdAbs demonstrated receptor-mediated brain uptake, enabling the pharmacologically effective delivery of non-permeable neuroactive drugs to the brain parenchyma.

Although antibodies have been extensively developed over the past decades, particularly for brain-related diseases, several limitations affect their application. These limitations include their large molecular size (approximately 150 kDa), high binding affinity to receptors involved in RMT, and efflux pumps mediated via the neonatal Fc receptor (FcRn) [119].

The neonatal fragment crystallizable (Fc) receptor, commonly called FcRn, is encoded by the *FCGRT* gene in humans. Functionally, FcRn is structurally like the MHC class I molecule, and it is associated with beta-2-microglobulin [120,121]. It plays a vital role in regulating the turnover of immunoglobulin G (IgG) and serum albumin [120]. The FcRn, predominantly found in endothelial and myeloid cells, helps recycle immunoglobulin G (IgG) and prolongs its half-life [122]. The BBB limits the transport of IgG from the blood to the brain but allows its swift efflux from the brain to the blood via reverse transcytosis after intracerebral injection. This IgG transport system at the BBB has properties consistent with the FcRn [123]. Unmodified IgG-based antibodies performed a less than 0.03% drug accumulation in the brain after 2 h of intravenous administration based on two similar studies [109,124]. In one of the studies, two different-sized bispecific antibodies (58 vs. 210 kDa) were designed with or without the IgG domain, and their brain pharmacokinetics were evaluated [124]. The smaller molecule demonstrated faster clearance from the bloodstream, a higher parenchymal-to-capillary concentration ratio, and earlier net elimination from the brain following injection compared to the larger molecule [124]. By reducing the binding affinity to FcRn, a bispecific anti-TfR and anti-Aβ antibody exhibited an enhanced Aβ clearance ability and BBB transportation ratio [125].

While molecular size is a critical factor for crossing the blood–brain barrier, the high binding affinity of antibodies to receptors presents a double-edged sword in BBB delivery. Strong receptor binding on the luminal side can impede dissociation from receptors at the abluminal membrane, thereby affecting efficient transcytosis [33]. A lower affinity increases the likelihood of the antibody dissociating from its targeting receptor. For example, a TfR-low but BACE1-high binding affinity bispecific antibody was developed by Genentech Inc. to clean the Aβ for Alzheimer’s disease [126]. The lower-affinity anti-TfR antibody variant was more effectively released from BBB, exhibited enhanced uptake into the brain, and demonstrated broader distribution within brain tissues [126]. It should also be mentioned that the low affinity reduces the construct’s binding to BCECs, increasing the likelihood of nonspecific uptake via peripheral organs. Other macromolecules, like peptides and aptamers with high-targeting specificity, may be another option for BBB-crossing delivery.

### 4.2. Peptides

Compared to large-sized antibodies, peptides with low molecular weights (500–5000 Da) exhibit high target selectivity while offering superior tissue penetration capabilities [127]. Peptides can form different secondary structures (α-helices, β-sheets, hairpins, and random coils). These structures are stabilized by inside hydrogen bonds, electrostatic and hydrophobic interactions, disulfide bonds, and cyclization [128]. Beyond their natural secondary structures, peptides are relatively easy to synthesize and can be artificially designed into cyclic or tricyclic structures at specific sites to increase binding affinity and stability [129].

Most binding peptides were identified from natural peptide toxins or phage display. The T7 peptide (HAIYPRH) has been widely used as a ligand targeting TfR [130]. A T7 peptide conjugated doxorubicin conjugates has been developed and performs in vitro antitumor activity [131]. The T7 peptide and its stabilized retro-inverso isoform ^D^T7 have been conjugated to various liposomes, nanoparticles, and cell membrane-coated nanoplatforms for glioma treatment, enhancing their ability to penetrate the blood-brain barrier [132,133,134,135]. Another 12-amino acid peptide (THRPPMWSPVWP) has also been developed to bind internally to the human TfR [136]. The TfR binding peptide was conjugated with (^68^)Ga to serve as a drug transport vehicle [137]. Additionally, it was conjugated to gold nanoparticles with another functional peptide, CLPFFD, to facilitate accumulation in the brain and target the toxic aggregates of β-amyloid for the treatment of Alzheimer’s disease [138]. A cationic liposome was conjugated with the 12-amino acid peptide ligand and carries the P-gp knockdown CRISPR/Cas9 plasmid to overcome brain pharmacoresistance [139].

Protein-miniaturized peptide is another source for targeting peptide identification. With the help of computer-aided peptide design technology, Ruan et al. designed a 12-AA peptide RAP12 from the 39 kDa RAP protein [140]. The RAP12 peptide (EAKIEKHNHYQK), containing key lysines (K253, K256) essential for LRP1 binding, enhances BBB crossing when modified onto drug delivery systems [140]. The cationic peptide Angiopep-2 (A2, TFFYGGSRGKRNNFKTEEY) is divided from a Kunitz-type domain protein, and it serves as a substrate for LRP1-mediated transcytosis, similar to aprotinin, amyloid precursor protein, and tissue factor pathway inhibitor [141]. Based on this, peptide-drug conjugate (PDC) ANG1005, a novel taxane derivative conjugated drug between A2 peptide with three paclitaxel molecules, exhibited an improved brain uptake capacity and antitumor efficacy [142]. In a following phase II study in patients with measurable recurrent brain metastases from breast cancer (NCT02048059) completed in 2020, ANG1005 treatment provided significant benefits for both CNS and systemic disease, even in patients previously treated with taxanes [143]. An open-label Phase III study (NCT03613181) is now comparing ANG1005 with Physician’s Best Choice in HER2-negative breast cancer patients with newly diagnosed leptomeningeal carcinomatosis and previously treated brain metastases. Further, A2 peptide-based PDC drugs like ANG1007 (A2-doxorubicin conjugate) and ANG1009 (A2-etoposide conjugate) were developed for the treatment of primary and secondary brain cancers [144]. Anti-HER2 mAb was also conjugated with A2 peptide to cross the BBB [145]. The AN2-mAb conjugate (ANG4043) binds to the LRP1 ECD and demonstrates potent antitumor efficacy against HER2-positive intracranial tumors in mice [145]. A similar strategy has been applied via a fusion AN2 peptide with anti-VEGF scFab for glioblastoma treatment [146]. Based on phage display technology, another LRP1 binding peptide, L57, with the sequence TWPKHFDKHTFYSILKLGKH, was developed with a binding affinity around 45 nM as the potential CNS-related drug delivery agent [147]. A cyclic peptide (KS-487) was also developed with higher LRP1 binding affinity, higher plasma stability, and better BBB permeability based on a structure–activity relationship study [148].

While no peptides have been designed explicitly for BBB shuttle via IGF1R/IGF2R targeting, some peptides identified for other diseases could be repurposed as delivery ligands due to their affinity for IGF1R or IGF2R. A research team from Nankai University recently screened a peptide fragment of IGF1 named IGF1C. The IGF1C peptide can inhibit the IGF1R in human cells and exhibit therapeutic efficacy for treating abdominal aortic aneurysm (AAA) in a rat and minipig model [149]. As the biomarker on the activated hepatic stellate cells (HSCs) for liver fibrosis, IGF2R-targeting peptides have also been developed as the ligand for HSC-related drug delivery [150]. Our group has screened several peptides via 5 rounds of protein-cell-based biopanning selection and identified Peptide-431 (VHWDFRQWWQPS), which can bind to the ECD domain of IGF2R [151]. Notably, based on the symmetrical structure of IGF2R, a dimerization-modified Peptide-431 performed a 9-fold increased binding affinity than monomeric peptide on IGF2R-expressed HSCs [151]. This strategy may be applied to other similar structure receptor-targeting peptide development, like that of the prostate-specific membrane antigen (PSMA) targeting peptides [152].

A hormone-like peptide encoded by esophageal cancer-related gene 4 (*Ecrg4*) is thought to play a role in various physiological processes [153]. Retrovirus-mediated expression cloning identified LOX-1 as a receptor for the peptide Ecrg4(71–132), with other scavenger receptors including SR-B2. The Ecrg4 peptide and SR-B2 interaction can be attenuated using the scavenger receptor inhibitor polyinosinic acid. This peptide, derived from a protein source, could potentially serve as a BBB shuttle by targeting scavenger receptors on BCECs [153].

Despite their advantages as delivery ligands or transport shuttles, inherent defects like short half-lives, which are easily hydrolyzed by enzymes during blood circulation or in cell plasma, limit the application of peptides in the clinic. To overcome this limitation, methods including switching the L-form to the D-form, backbone modification, PEGylation, lipidation, N-methylation, stapled modification, and cyclic modification have been applied to develop therapeutic peptides [154,155].

### 4.3. Aptamers

Aptamers are short, single-stranded DNA or RNA oligonucleotides with a three-dimensional structure that bind specific targets with a high affinity and specificity [156]. Their synthesis is achieved through an in vitro evolution process known as the systematic evolution of ligands by exponential enrichment (SELEX) [157]. The strategy was developed thirty-five years ago and focused on the iterative selection of high-affinity nucleic acid ligands through three essential steps: first, incubating a randomized oligonucleotide library with the chosen target; second, separating the bound oligonucleotides from those that remain unbound; and finally, recovering and amplifying the target-bound DNAs or RNAs for subsequent selection cycles [158,159]. Due to their small size, aptamer oligonucleotides efficiently penetrate tumor cells and can be functionalized with various biomolecules, making them ideal for sensing, imaging, and targeted drug delivery [160]. Some peptides were designed to target BBB endothelial cells or membrane proteins, enabling drug transport across the BBB, and they have been summarized in a previous review [161].

Numerous aptamers have been engineered to target the TfR, facilitating targeted drug delivery and cancer therapy applications [162]. A notable example is the HG1–9 DNA aptamer, developed via cell-SELEX, which binds the human TfR with high affinity [162]. This aptamer can traverse the epithelial barrier through TfR-mediated transcytosis, positioning it as a promising candidate for targeted cancer diagnostics and therapeutics. Another E3 aptamer has been developed that utilizes the TfR pathway for internalization into cancer cells rapidly [163]. Additionally, the E3 aptamer can deliver highly cytotoxic drugs to cancer cells as aptamer-highly toxic drug conjugates (ApTDCs), thus enhancing the efficacy of targeted cancer therapy. Such TfR-binding aptamers can be further used to form ApTDCs for brain diseases. Choi et al. further identified several aptamer nanoconstructs via microphysiological system-based SELEX technology under human physiological conditions as BBB shuttle for brain drug delivery [164].

Ryu et al. recently identified a conformation-specific allosteric IR-targeting aptamer named IR-A43 [165]. The IR-A43 aptamer functions as a positive allosteric modulator, enhancing receptor activation by stabilizing the binding of a ligand to residue Q272 within the cysteine-rich domain of IGF2R. While IR-A43 is inactive on its own, it amplifies insulin-induced autophosphorylation and downstream signaling of the insulin receptor when insulin is present [165]. The IR-A62 aptamer, developed by the same research group, is a unique modulator of the IR that functions as a biased agonist, selectively inducing monophosphorylation at Y1150 of the receptor. IR-A62 acts as a positive allosteric modulator (PAM-agonist) at low concentrations, enhancing insulin binding. However, it transitions to a negative allosteric modulator (NAM-agonist) at high concentrations, competing with insulin for receptor binding [166,167]. An IGF2R-specific targeting aptamer was developed via SELEX for HSC targeting [168]. Aptamer-20 demonstrated a dissociation constant (Kd) of 35.5 nM for the ECD of IGF2R and exhibited high affinity for IGF2R-expressing cells, with a Kd of 45.12 nM. It was engineered into a siRNA-aptamer chimera with anti-fibrotic siRNA, enabling targeted delivery to HSC cells and effectively triggering gene silencing activity. In vivo biodistribution studies of the siRNA-aptamer chimera revealed high and specific liver uptake in rats with CCl4-induced liver fibrosis, demonstrating its ability to transport biomolecule drugs by targeting IGF2R [168].

Like peptides, aptamers face inherent challenges such as serum instability and renal filtration, which limit their applications. However, advancements in RNA-based therapies (such as siRNA and mRNA) have led to the widespread use of 2′-fluoronucleotide and 2′-O-methylnucleotide modifications to enhance the stability of aptamer backbones. Other chemical modifications like PEGylation have also been employed to improve aptamer performance [169].

## 5. RMT-Driven Delivery Systems for Brain Diseases

Nanomedicine exhibits unique advantages in drug delivery and has been explored in several therapeutics, including CNS diseases [170]. Due to the size constraints of nanoparticles at the nanoscale, most functional nanomedicine designed for treating brain diseases relies on RMT to cross the BBB, enabling the targeted accumulation at pathological sites in the brain [171].

Since the approval of Doxil (doxorubicin liposome) by the FDA in 1995 as the first liposome-based drug for cancer treatment, several other liposomal formulations have entered the market, including Onivyde (irinotecan liposome), Vyxeos (daunorubicin and cytarabine liposome), and Marqibo (vincristine liposome) [172,173,174,175]. Although no liposome-based drugs have been explicitly approved for CNS disorders, numerous liposomal formulations targeting brain diseases have been developed and discussed in a prior review [176]. According to data from ClinicalTrials.gov, most liposome-based drugs in clinical trials are focused on applications such as treating brain metastases from breast cancer or glioblastoma as extensions of their approved indications. Notably, a phase I safety study is underway for ADx-001, a novel intravenously administered, gadolinium-containing, molecularly targeted liposomal product. This study (NCT05453539) aims to evaluate the proof-of-concept for a new imaging diagnostic in patients with suspected Alzheimer’s disease. Zhao et al. recently developed a polymer-locking fusogenic liposome for GBM siRNA delivery via surface modification with Angiopep-2 [177]. Designed with a reactive oxygen species (ROS)-cleavable linker, the polymer-locking fusogenic liposome initiated fusion only after penetrating the BBB and encountering high ROS levels within GBM tissue, exhibiting an effective RNAi therapy for CNS treatment [177].

Lipid nanoparticles (LNP) have also been applied as the BBB-crossing drug carrier for brain diseases [178]. As the first approved siRNA delivery system in 2018, Onpattro demonstrated the remarkable potential of lipid nanoparticles (LNPs) in genomic medicine. This potential was further highlighted by the successful use of LNP-based COVID-19 mRNA vaccines developed by Pfizer-BioNTech and Moderna in 2020 [179]. Based on these, LNP may be more suitable for siRNA therapy for CNS diseases. A lipid nanoparticle (DAT-LNP) with a dual-functional peptide enables glioma-targeted immunotherapy by crossing the BBB has been developed [180]. It facilitates brain tissue accumulation and activates immune responses by maturing dendritic cells, polarizing M1 macrophages, and stimulating CD8+ T cells [180]. By targeting the LRP-1, an enzyme-sensitive LNP was designed to deliver siRNA for GBM treatment [181]. The positively charged LNPs are masked with a negatively charged, PEGylated cleavable lipopeptide recognized by matrix metalloproteinases (MMPs). A charge switch is triggered upon proteolytic cleavage, enhancing cellular uptake and siRNA release for effective gene silencing [181]. A dual-ligand functionalized lipid nanoparticle (AM31 LNPs) has been recently developed as a promising vehicle for RNA therapeutics, specifically targeting microglia and astrocytes in neural disorders [182].

Another widely utilized nanosized drug delivery system is polymicelle [183]. Conjugating receptor-targeting ligands to the surface of micelles enhances their ability to cross the BBB for CNS-related drug delivery [184]. A transferrin-modified glutathione (GSH)-sensitive hyaluronic acid derivative micelle has been developed to deliver HSP90 inhibitors, aiming to enhance the therapeutic efficacy of brain cancers [185]. In another study, the TfR-binding T12 peptide was modified to the PEG-PLA polymer micelle to deliver paclitaxel for GBM [186]. The TfR-T12-PEG-PLA/PTX polymeric micelles can effectively cross the BBB and target gliomas, demonstrating their potential to improve therapeutic outcomes in glioblastoma multiforme [186]. Similarly, Angiopep-2-modified PE-PEG-based polymeric micelles have been developed for the targeted delivery of amphotericin B to the brain [187]. The same Angiopep-2-modified polymeric micelle has also been used to treat intracranial fungal infection [188].

Poly (lactic-co-glycolic acid) (PLGA) is a biodegradable and biocompatible polymer used in several FDA-approved products [189]. PLGA has been used for drug delivery with modifications like mAb, peptide, and aptamer for different diseases [190,191]. A Tf-coated PLGA nanoparticle exhibited a higher brain accumulation for siRNA delivery in traumatic brain injury [192]. PLGA nanoparticle-based formulations for crossing the blood–brain barrier and drug delivery have been extensively reviewed in previous studies [193,194]. Other functional polymers with different properties were designed and applied for CNS treatment. In recent studies, a polyethyleneimine (PEI) nanocomplex conjugated with neuropilin-1 (NRP-1) targeting peptide and vascular endothelial growth factor receptor 2 (VEGFR-2) binding peptide demonstrated significant efficiency in crossing the blood–brain barrier (BBB) and targeting glioma tissue in vivo [195]. Galactose-modified poly(ethylene glycol)-*block*-poly[(*N*-(3-methacrylamidopropyl)] guanidinium [Gal-PEG-*b*-P(Gu)] was designed and synthesized to carry siRNA for Alzheimer’s disease therapy via the GLUT1-mediated RMT [65].

Microbubbles (MBs) in ultrasound imaging and drug delivery are typically spherical [196]. When tagged with an anti-TfR antibody, a research team from Aachen University demonstrated, polymer-based rod-shaped MBs exhibited better binding to BBB endothelium and improved drug delivery efficiency compared to their spherical counterparts via the higher available surface for interaction. This highlights the potential of antibody-modified, nonspherical MBs for targeted brain drug delivery [197]. In another study, apolipoprotein E receptor-binding peptide was modified to enhance MBs’ crossing of the BBB under low-energy ultrasound [198].

## 6. Novel Strategy for Brain-Related Diseases via RMT

### 6.1. Viral Vectors

For over two decades, viral vectors have been explored for gene delivery in neurological disorders due to their high transfection efficiency [199]. Despite success with lentivirus, herpes simplex virus, adenovirus, and adeno-associated virus (AAV) vectors, clinical applications face challenges like complex manufacturing, high costs, and safety concerns [200]. AAV vectors, notable for their firm safety profiles and effective brain gene delivery, are prominent in clinical trials [201]. However, crossing the BBB remains challenging, often requiring invasive methods like stereotaxic or cerebrospinal fluid injections. While new viral vectors show promise, most rely on direct brain injection [202]. In 2024, an engineered AAV capsid was generated with the ability to the human TfR1 on the BBB [203]. The reprogrammed AAV capsid (BI-hTFR1) showed significant CNS-specific reporter expression, 40 to 50 times greater than AAV9, in human TFRC knockin mice. This demonstrates BI-hTFR1’s potential as a vector for CNS gene therapy [203].

### 6.2. Exosomes

Exosomes, small extracellular vesicles, offer notable advantages over synthetic nanoparticles due to their non-immunogenic nature, allowing them to circulate stably and for prolonged periods in the bloodstream [204]. Exosomes derived from BCECs mediate molecular exchange across the BBB and enhance intercellular communication in the brain [205]. Engineered exosomes with functionalized targeting ligands have shown a potential to strengthen their ability to cross the BBB significantly. These modifications improve targeting specificity and facilitate efficient delivery of therapeutic agents to the brain, overcoming one of the major challenges in neurological drug delivery. Kim et al. conjugated the TfR1-binding T7 peptide into the exosome for GBM treatment [206]. Systemic delivery studies of T7-peptide decorated exosomes (T7-exo) carrying microRNA-21 antisense oligonucleotides were conducted in intracranial glioblastoma rat models through intravenous tail vein injection. T7-exo demonstrated higher efficiency in delivering microRNA-21 antisense to the brain, reducing miR-21 levels in glioblastoma and leading to decreased tumor sizes [206].

### 6.3. Boron Neutron Capture Therapy (BNCT)

Boron neutron capture therapy (BNCT) is a non-invasive treatment modality that uses the selective destruction of tumor cells through high-energy alpha particles generated via the neutron capture reaction of boron [207]. This technique shows significant potential for treating recurrent tumors in previously irradiated areas and tumors located near critical structures like the brainstem and spinal cord [208]. In 2020, Japan approved BNCT as a new cancer radiation treatment for unresectable advanced or recurrent head and neck tumors [209]. Various boron-containing compounds have been developed for application in BNCT, including boronophenylalanine (BPA), sodium borocaptate (BSH), and boronated porphyrins. These compounds serve as boron delivery agents, enabling targeted accumulation in tumor tissues to enhance the therapeutic efficacy of BNCT [210]. Despite their potential as therapeutic agents, boron-containing compounds face significant challenges in treating brain tumors. The insufficient selective accumulation of boron drugs within tumor tissues, coupled with the limited penetration depth of thermal neutrons, are recognized as key factors contributing to the suboptimal therapeutic outcomes and associated adverse effects in BNCT [211]. Various boronated modifications have been developed to address these limitations, with some of these compounds advancing to clinical trials. These modifications enhance boron delivery, improve tumor selectivity, and optimize therapeutic efficacy [210]. As the first modified boronated drug, BPA demonstrates the ability to cross the BBB via the LAT1. Similarly, it is selectively taken up by GBM cells through LAT1, making it a promising agent for targeted delivery [212]. In addition to BPA, boron-10 (^10^B) has been further incorporated into various drug delivery systems designed to enhance its therapeutic efficacy in BNCT. Initially developed for other CNS diseases, these delivery platforms can improve boron targeting, optimize drug biodistribution, and increase BNCT treatment efficiency [211].

Other non-invasive strategies, including photodynamic and sonodynamic therapies, have also been developed for CNS diseases relying on RMT and summarized in previous reviews [213,214]. A γ-Glutamyl transpeptidase (GGT)-activable nanoprobe has been developed for the immuno-sonodynamic therapy of glioma [215]. With the guidance of the ApoE-binding peptide, the nanoprobe crosses the BBB and swells to release a sonosensitizer and immune agonist under ultrasound, inducing a robust anticancer immune response [215]. With the advent of engineered high-targeting, high-affinity biomacromolecules, integrating these targeted macromolecules with innovative therapeutic strategies enables their effective accumulation at brain lesion sites. These synergistic approaches harness the intrinsic advantages of novel therapies, thereby enhancing their potential for clinical translation and maximizing therapeutic efficacy.

## 7. Conclusions

Receptor-mediated transcytosis represents a transformative approach to overcoming the blood–brain barrier, offering a pathway for efficient and targeted drug delivery to the central nervous system. By developing macromolecular ligands such as antibodies, peptides, and aptamers, significant progress has been made in enhancing the specificity and efficiency of brain drug delivery systems. These advances hold great promise for treating a range of brain-related diseases, from neurodegenerative disorders to malignancies. Furthermore, emerging strategies leveraging RMT, including viral vectors, exosomes, and boron neutron capture therapy, open new avenues for precision medicine in the CNS. While challenges remain, such as optimizing ligand-receptor interactions, ensuring drug stability, and minimizing off-target effects, ongoing research continues to refine these approaches.

By integrating conventional and innovative methodologies, RMT-driven delivery systems have the potential to revolutionize the treatment of brain diseases. The continued exploration and development of these systems will likely lead to breakthroughs that address unmet clinical needs, ultimately improving outcomes for patients with CNS disorders or brain tumors.

## Figures and Tables

**Table 1:** Approved drugs for CNS diseases.

Drug Name	Target	Function	Molecular Weight (Da)	Primary Treatment Diseases	Brain-Related Diseases	First Approved Year
Lecanemab	Beta-amyloid	Removes beta-amyloid plaques in Alzheimer's disease	147k	Alzheimer's disease	Alzheimer's disease	2023
Aducanumab	Amyloid-beta plaques	Reduces amyloid plaques in Alzheimer's disease	145k	Alzheimer's disease	Alzheimer's disease	2021
Naxitamab	GD2 ganglioside	Targets GD2 in high-risk pediatric neuroblastoma	144k	Neuroblastoma	Neuroblastoma	2020
Dinutuximab	GD2 ganglioside	Targets GD2 in high-risk pediatric neuroblastoma	144k	Neuroblastoma	Neuroblastoma	2015
Bevacizumab	VEGF (vascular endothelial growth factor)	Inhibits tumor blood vessel growth in glioblastoma	149k	Metastatic colorectal cancer	Glioblastoma	2004
Tovorafenib	BRAF (v-raf murine sarcoma viral oncogene homolog 1)	Inhibits BRAF in melanoma and brain tumors	506.29	Melanoma	Brain metastases	2024
Vorasidenib	Mutated IDH1/IDH2 genes	Inhibits mutated IDH genes in gliomas	414.74	Astrocytoma, Oligodendroglioma	Astrocytoma, Oligodendroglioma	2024
Belzutifan	Hypoxia-inducible factor 2α (HIF-2α)	Inhibits HIF-2α in renal cell carcinoma	383.34	Renal cell carcinoma	Brain metastases	2021
Trametinib	MEK (mitogen-activated protein kinase)	Inhibits MEK signaling in brain tumors	615.404	Brain tumors	Brain tumors	2013
Dabrafenib	BRAF (v-raf murine sarcoma viral oncogene homolog 1)	Inhibits BRAF in melanoma and brain tumors	519.56	Melanoma	Brain metastases	2013
Everolimus	mTOR (mammalian target of rapamycin)	Inhibits mTOR signaling in brain tumors	958.24	Advanced kidney cancer	Brain tumors	2009
Temozolomide	DNA	Alkylates DNA in various brain tumors	194.154	Brain tumors	Brain tumors	1999
Eflornithine Hydrochloride	Ornithine decarboxylase	Inhibits ornithine decarboxylase in brain tumors	236.64	Neuroblastoma	Neuroblastoma	2000
Procarbazine, Lomustine, Vincristine (PCV)	DNA and microtubules	Combination chemotherapy for brain tumors	N/A	Brain tumors	Brain tumors	1976
Carmustine	DNA	Alkylates DNA in brain tumors	214.05	Brain tumors	Brain tumors	1977

N/A: Not applicable.
